# Association between Official Development Assistance for Water and Sanitation and Countries’ Needs from 2002 to 2019

**DOI:** 10.3390/ijerph191711134

**Published:** 2022-09-05

**Authors:** Sumin Kim, Seungman Cha, Yan Jin

**Affiliations:** 1 Department of Global Development and Entrepreneurship, Graduate School of Global Development and Entrepreneurship, Handong Global University, Pohang 37554, Korea; 2 Department of Clinical Research Design and Evaluation, Samsung Advanced Institute for Health Sciences & Technology (SAIHST), Sungkyunkwan University, Seoul 06351, Korea; 3 Department of Pediatrics, Samsung Medical Center, Sungkyunkwan University School of Medicine, Seoul 06351, Korea; 4 Department of Microbiology, Dongguk University College of Medicine, Gyeongju 38066, Korea

**Keywords:** official development assistance, water, sanitation, coverage

## Abstract

Although an enormous amount of aid has been invested in water and sanitation, few studies have analyzed the criteria used by the international community to select recipient countries and allocate official development assistance (ODA). We examined whether ODA has been allocated in proportion to water and sanitation needs and whether this has improved over the past 18 years. For water, 141 low- and middle-income countries (LMICs) and least-developed countries (LDCs) were selected, whereas 104 LMICs and LDCs were chosen for sanitation due to a lack of data. For aid disbursement, we used ODA data from the Organization for Economic Cooperation and Developments (OECD) Creditor Reporting System (CRS) from 2002 to 2019. OECD CRS data on water and sanitation are available from 2002 onward. For water and sanitation coverage, we collected data from the WHO/UNICEF Joint Monitoring Program from 2002 to 2019. We examined annual ODA trends and performed linear regression analysis adjusted for GNI per capita using log-transformed dependent variables. Neither total ODA nor ODA per capita was found to be associated with countries’ needs for water and sanitation. For instance, no significant association was detected between at least basic water and sanitation coverage and total ODA per capita in 2019 (log coefficient: 0.002, *p* = 0.52). The global community needs to determine the reasons for and means of addressing this discrepancy.

## 1. Introduction

Global access to safe drinking water and sanitation is a basic right, and its effects go well beyond health improvements to human dignity and well-being, women’s privacy, and children’s education [1,2]. The global health community highlights the importance of universal and equitable access to drinking water and sanitation and emphasizes the importance of achieving safely managed water and sanitation [3,4]. At the same time, researchers have paid attention to wastewater treatment in recent years [5,6].

Sustainable development goals (SDGs) focus on eradicating inequality and vulnerability [3]. Lack of access to safe water and sanitation services results in diseases such as diarrhea, dysentery, polio, and typhoid [7]. Of particular note, improving water and sanitation is critical for reducing under-five mortality [8,9]. In 2019, 5,300,000 children in this age group died, and diarrhea and infectious diseases accounted for 9.1% and 49.2% of these deaths, respectively [4]. Worldwide, 443 million school days are lost annually due to water-related diseases and infections caused by inadequate sanitation [2,10].

The global health community has witnessed steady improvements between 2015 and 2020, during which time access to basic drinking water increased from 71% to 74%, and basic sanitation services increased from 45% to 48% in developing countries [11]. Meanwhile, the total official development assistance (ODA) for water and sanitation increased at an annual rate of 5% from 2006 to 2016, with an average commitment of 14.3 billion in 2015. Moreover, 2.3 billion people gained access to drinking water from improved water sources, and 2 billion people experienced improved sanitation from 1990 in developing countries [12,13].

Despite these advances, water and sanitation coverage are far from universal. By 2030, the coverage of safely managed drinking water is expected to reach 81%, which means 1.6 billion people will lack access to a safe water supply. Moreover, safely managed sanitation coverage is projected to reach 67% but be unavailable to 2.8 billion people across the globe [11]. In 2020, it was estimated around 1.4 billion people across the globe live in areas of high water vulnerability. In the same year, one in three people suffered due to a lack of safely managed water, and two in five people lacked access to a basic sanitation facility [11,14].

Globally, ODA in water and sanitation substantially decreased in 2020 compared to the previous year (USD 3567 million in 2020 vs. USD 4109 million in 2019), although the attention of the global community and many country leaders seems to have been drawn to the importance of water and sanitation to tackle the spread of COVID-19 [15,16,17]. Accordingly, access to clean water, sanitation, and hygiene (WASH) is becoming a more important priority. Furthermore, improved drinking water and sanitation coverage in low- and middle-income countries is an effective means of reducing the risk of global pandemics. In addition, the global health community has used improved access to safe drinking water and sanitation as an indicator of poverty reduction [18]. Nonetheless, inequalities in access to clean water and sanitation persist, although universal health coverage (UHC) is at the core of sustainable development goals (SDGs) [19,20,21]. Achieving UHC by 2030 has been predicted to require a four-fold increase in the pace of providing safely managed drinking water and sanitation services [11].

Development assistance for water and sanitation coverage requires more attention to achieve these goals. Studies on ODA have focused on assessments of performance and impacts on health, and few have investigated whether aid allocation for water and sanitation coverage meets the needs of recipient countries [7,22,23,24]. We aimed to assess associations between the amount of ODA provided and the water and sanitation needs of recipient countries over the period 2002–2019. The present study summarizes ODA targeting for water and sanitation coverage over this period [25].

## 2. Materials and Methods

### 2.1. Target Countries

We analyzed 141 LMICs and LDCs for water and 104 LMICs and LDCs for sanitation. For this study, we restricted the countries to those developing countries for which we could identify the annual ODA amount from the Organization for Economic Co-operation and Development (OECD) creditor responding system (CRS) database, as well as coverage of water or sanitation from the WHO/UNICEF Joint Monitoring Programme (JMP) report. For the definition of LMICs and LDCs, we referred to the categories of the OECD. A full list of targeted countries is provided in Table S1.

### 2.2. Data Sources and Definition

We collected data from the 141 and 104 countries’ profiles in the CRS of OECD Development Assistance Committee (DAC) disbursement database from 2002 to 2019 [15]. OECD CRS data on water and sanitation are available from 2002 onward.

Disbursement records were collected according to CRS object codes, i.e., “140-I.4: water supply and sanitation, total”, “14030: basic drinking water supply and basic sanitation”, “14031: basic drinking water supply”, and “14032: basic sanitation”. Data for CRS code 140 were collected from 2002 to 2019, and data for CRS codes 14030, 14031, and 14032 were collected from 2010 to 2019 because these data were unavailable prior to 2010.

The definitions of water and sanitation coverage are different between the MDG and SDG periods. Still, the JMP group published data on each type of coverage based on the new definitions dating back to 2000. We applied the updated definitions in this study. For data on water and sanitation coverage, we referred to WHO/UNICEF Joint Monitoring Programme (JMP) records from 2002 to 2019 [26]. Safely managed drinking water is defined as improved water that can be used when needed at home and is free from contamination (feces and chemicals). The definition of basic drinking water requires that it takes less than 30 min (round-trip, including waiting time) to obtain drinking water [27]. Safely managed sanitation is defined as access to improved facilities, not shared with other households, that safely dispose of excrement or access to external facilities. Basic sanitation for improved facilities also includes not sharing with other households [28].

GNI per capita data in the World Bank and gross national income were converted to 2019 US dollars and international dollars using the World Bank Atlas method and purchasing power parity [29]. Total population data were collected from the World Bank [30].

### 2.3. Data Analysis

Initially, we examined trends in the annual ODA growth for water and sanitation by tracking annual ODA disbursements to 141 LMICs and LDCs for water and 104 LMICs and LDCs for sanitation from 2002 to 2019. We also estimated the annual growth rate of ODA per capita/annum by dividing total ODA by the total population.

We investigated whether ODA for water and sanitation was disbursed according to needs in terms of coverage. In this study, needs were defined as the proportion of people who did not have access to water or sanitation services by each type of the JMP definitions. Hence, needs were defined as 100% minus water or sanitation coverage, respectively. In the analysis, we maintained the use of coverage rather than generating a new variable for needs. We did not use the number of people without water or sanitation because this could cause potential bias insofar as populous countries with higher coverage would have more needs than countries with smaller populations but lower coverage.

After performing log transformation on dependent variables—that is, total water supply and sanitation (CRS code 140-I.4) from 2002 to 2019, a combination of basic drinking water supply (14031) and basic drinking water supply and basic sanitation (14030) from 2010 to 2019, and a combination of basic sanitation (14032) and basic drinking water supply and basic sanitation (14030) from 2010 to 2019—ordinary least squares regression with adjustment for GNI per capita was performed between independent and dependent variables. When the dependent variable was ODA per capita for total water supply and sanitation (CRS code 140-I.4), we used (1) at least basic drinking water and sanitation services coverage, or (2) safely managed drinking water and sanitation services coverage as independent variables. When the dependent variable was ODA per capita for basic drinking water supply, the independent variables were (1) at least basic drinking water coverage, or (2) safely managed drinking water coverage. Third, when the dependent variable was ODA per capita for basic sanitation, the independent variables were (1) at least basic sanitation coverage or (2) safely managed sanitation coverage. Correlation analysis was conducted as a supplementary analysis.

In addition, we also explored whether cumulative amounts of ODA per capita for water and sanitation in 2016–2019 were associated with needs in 2015 using the three sets of dependent and independent variables. STATA version 16.0 was used for the analysis.

## 3. Results

**Figure 1:**
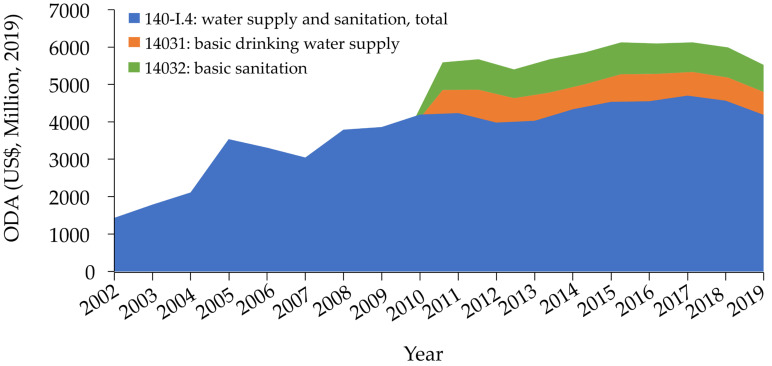
Official development assistance for water and sanitation, stacked area chart, 2002–2019. (y-axis units: millions USD (2019), ODA = official development assistance). There are no data for “ODA for basic drinking water supply” or “ODA for basic sanitation” before 2010.

Table 1 presents trends in ODA and ODA per capita and annual ODA growth rates. Total ODA for water and sanitation increased over time from 2002 but has shown a slight decrease since around 2016. ODA for basic water and sanitation increased from 2010 and decreased from 2017, and the amount of aid was lower in 2019 than in 2011 (Figure 1).

**Figure 2:**
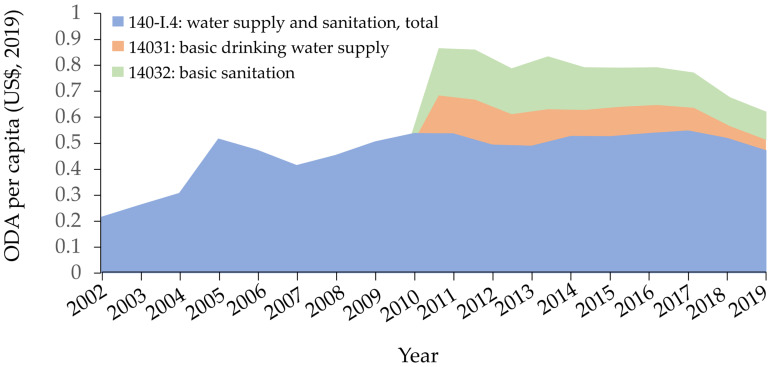
Official developments assistance per capita for water and sanitation, stacked area chart, 2010–2019. (y-axis units: millions USD (2019), ODA per capita = official development assistance per capita). There are no data for “ODA for basic drinking water supply” or “ODA for basic sanitation” before 2010.

Figure 1 shows that ODA for water and sanitation rapidly increased from 2004 to 2005 and remained stagnant from 2017. ODA for basic water and sanitation increased between 2012 and 2013 but started to decline in 2016. Figure 2 shows ODA per capita for water and sanitation changed slightly and that ODA per capita for basic water and sanitation did not increase from 2010.

**Figure 3:**
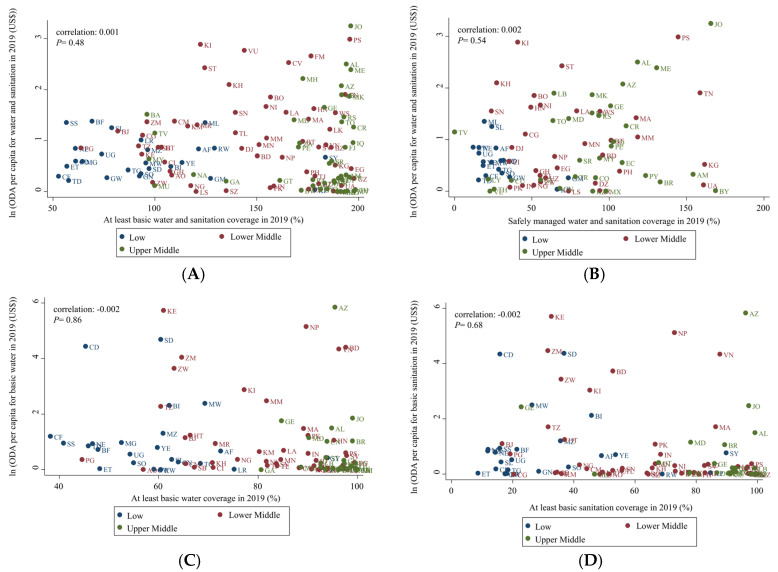

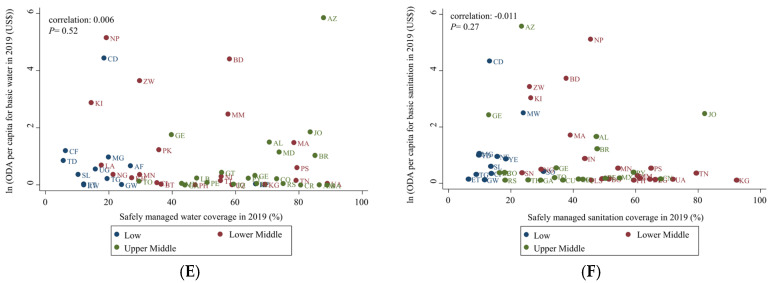
Associations between ODA for water and sanitation and coverage in 2019 (x-axis: water and sanitation and water or sanitation coverage (%), y-axis: ODA per capita (USD$), log transformation, income groups (colored) were classified as recommended by the World Bank. (**A**) ODA per capita for water and sanitation for at least basic water and sanitation coverage in 2019, (**B**) ODA per capita for water and sanitation for safely managed water coverage in 2019, (**C**) ODA per capita for basic water for at least basic water coverage in 2019, (**D**) ODA per capita for basic sanitation for at least basic sanitation coverage in 2019, (**E**) ODA per capita for basic sanitation for safely managed water coverage in 2019, (**F**) ODA per capita for basic sanitation for safely managed sanitation coverage in 2019. Afghanistan (AF), Albania (AL), Algeria (DZ), Angola (AO), Argentina (AR), Armenia (AM), Azerbaijan (AZ), Bangladesh (BD), Belarus (BY), Belize (BZ), Benin (BJ), Bhutan (BT), Bolivia (BO), Bosnia and Herzegovina (BA), Botswana (BW) Brazil (BR), Burkina Faso (BF), Burundi (BI), Cabo Verde (CV), Cambodia (KH), Cameroon (CM), Central African Republic (CF), Chad (TD), China (CN), Colombia (CO), Comoros (KM), Congo (CG), Costa Rica (CR), Côte d’Ivoire (CI), Cuba (CU), Democratic People’s Republic of Korea (KP), Democratic Republic of the Congo (CD), Djibouti (DJ), Dominica (DM), Dominican Republic (DO), Ecuador (EC), Egypt (EG), El Salvador (SV), Equatorial Guinea (GQ), Eritrea (ER), Eswatini (SZ), Ethiopia (ET), Fiji (FJ), Gabon (GA), Gambia (GM), Georgia (GE), Ghana (GH), Grenada (GD), Guatemala (GT), Guinea (GN), Guinea-Bissau (GW), Guyana (GY), Haiti (HT), Honduras (HN), India (IN), Indonesia (ID), Iran (IR), Iraq (IQ), Jamaica (JM), Jordan (JO), Kazakhstan (KG), Kenya (KE), Kiribati (KI) Kyrgyzstan (KG), Lao People’s Democratic Republic (LA), Lebanon (LB), Lesotho (LS), Liberia (LR) Libya (LY), Madagascar (MG), Malawi (MW), Malaysia (MY), Maldives (MV), Mali (ML), Marshall Islands (MH), Mauritania (MR), Mauritius (MU), Mexico (MX), Micronesia (FM), Moldova (MD), Mongolia (MN), Montenegro (ME), Morocco (MA), Mozambique (MZ), Myanmar (MM), Namibia (NA), Nepal (NP), Nicaragua (NI) Niger (NE), Nigeria (NG), North Macedonia (MK), Pakistan (PK), Panama (PA), Papua New Guinea (PG), Paraguay (PY), Peru (PE), Philippines (PH), Rwanda (RW), Samoa (WS), Sao Tome and Principe (ST), Senegal (SN), Serbia (RS), Sierra Leone (SL), Solomon Islands (SB), Somalia (SO), South Africa (ZA), South Sudan (SS), Sri Lanka (LK), Sudan (SD), Suriname (SR), Syrian Arab Republic (SY), Tajikistan (TJ), Tanzania (TZ), Thailand (TH), Timor-Leste (TL), Togo (TG), Tonga (TO), Tunisia (TN), Turkey (TR), Turkmenistan (TM), Tuvalu (TV), Uganda (UG), Ukraine (UA), Uzbekistan (UZ), Vanuatu (VU), Venezuela (VE), Viet Nam (VN), West Bank and Gaza Strip (PS), Yemen (YE), and Zambia (ZM).

Table 2 present the results of linear regression analysis between the amount of ODA and water and sanitation coverage. Figure 3 present the results of correlation analysis between the amount of ODA and water and sanitation coverage. The correlation analysis results for each year from 2002 to 2019 are included in Table S2.

The total ODA and ODA amounts per capita were not found to be associated with water and sanitation coverage. ODA per capita for water and sanitation in 2019 were not associated with at least water and sanitation coverage (coefficient: 0.001, *p* = 0.48). Likewise, for basic water and sanitation, no association was detected between ODA per capita and coverage during the 18-year study period.

**Figure 4:**
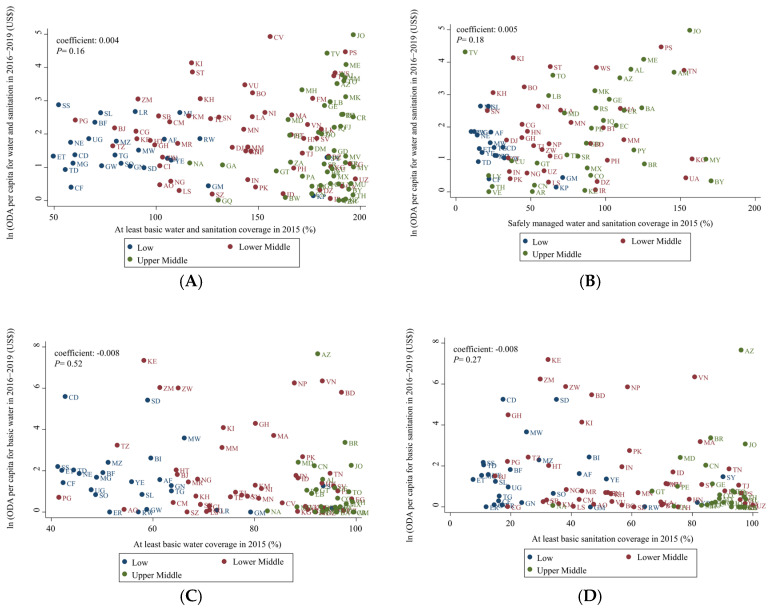

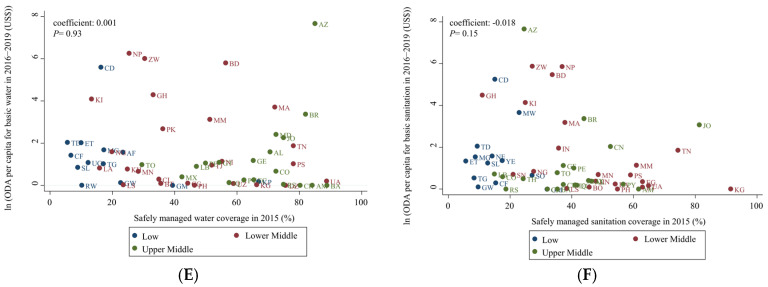
Association between ODA from 2016 to 2019 and water and sanitation coverage in 2015 (x-axis: water and sanitation and water or sanitation coverage (%), y-axis: ODA per capita (USD$), log transformation, income groups (colored) were classified as recommended by the World Bank. (**A**) ODA per capita for water and sanitation from 2016 to 2019 and at least basic water and sanitation coverage for 2015, (**B**) ODA per capita for water and sanitation from 2016 to 2019 and safely managed water and sanitation coverage for 2015, (**C**) ODA per capita for basic water from 2016 to 2019 and at least basic water coverage for 2015, (**D**) ODA per capita for basic sanitation from 2016 to 2019 and at least basic sanitation coverage for 2015, (**E**) ODA per capita for basic water from 2016 to 2019 and safely managed water coverage for 2015, (**F**) ODA per capita for basic sanitation from 2016 to 2019 and safely managed sanitation coverage for 2015. Afghanistan (AF), Albania (AL), Algeria (DZ), Angola (AO), Argentina (AR), Armenia (AM), Azerbaijan (AZ), Bangladesh (BD), Belarus (BY), Belize (BZ), Benin (BJ), Bhutan (BT), Bolivia (BO), Bosnia and Herzegovina (BA), Botswana (BW), Brazil (BR), Burkina Faso (BF), Burundi (BI), Cabo Verde (CV), Cambodia (KH), Cameroon (CM), Central African Republic (CF), Chad (TD), China (CN), Colombia (CO), Comoros (KM), Congo (CG), Costa Rica (CR), Côte d’Ivoire (CI), Cuba (CU), Democratic People’s Republic of Korea (KP), Democratic Republic of the Congo (CD), Djibouti (DJ), Dominica (DM), Dominican Republic (DO), Ecuador (EC), Egypt (EG), El Salvador (SV), Equatorial Guinea (GQ), Eritrea (ER), Eswatini (SZ), Ethiopia (ET), Fiji (FJ), Gabon (GA), Gambia (GM), Georgia (GE), Ghana (GH), Grenada (GD), Guatemala (GT), Guinea (GN), Guinea-Bissau (GW), Guyana (GY), Haiti (HT), Honduras (HN), India (IN), Indonesia (ID), Iran (IR), Iraq (IQ), Jamaica (JM), Jordan (JO), Kazakhstan (KG), Kenya (KE), Kiribati (KI), Kyrgyzstan (KG), Lao People’s Democratic Republic (LA), Lebanon (LB), Lesotho (LS), Liberia (LR), Libya (LY), Madagascar (MG), Malawi (MW), Malaysia (MY), Maldives (MV), Mali (ML), Marshall Islands (MH), Mauritania (MR), Mauritius (MU), Mexico (MX), Micronesia (FM), Moldova (MD), Mongolia (MN), Montenegro (ME), Morocco (MA), Mozambique (MZ), Myanmar (MM), Namibia (NA), Nepal (NP), Nicaragua (NI), Niger (NE), Nigeria (NG), North Macedonia (MK), Pakistan (PK), Panama (PA), Papua New Guinea (PG), Paraguay (PY), Peru (PE), Philippines (PH), Rwanda (RW), Samoa (WS), Sao Tome and Principe (ST), Senegal (SN), Serbia (RS), Sierra Leone (SL), Solomon Islands (SB), Somalia (SO), South Africa (ZA), South Sudan (SS), Sri Lanka (LK), Sudan (SD), Suriname (SR), Syrian Arab Republic (SY), Tajikistan (TJ), Tanzania (TZ), Thailand (TH), Timor-Leste (TL), Togo (TG), Tonga (TO), Tunisia (TN), Turkey (TR), Turkmenistan (TM), Tuvalu (TV), Uganda (UG), Ukraine (UA), Uzbekistan (UZ), Vanuatu (VU), Venezuela (VE), Viet Nam (VN),West Bank and Gaza Strip (PS), Yemen (YE), and Zambia (ZM).

Table 3 shows the results of regression analysis for the association between cumulative ODA per capita for water and sanitation in 2016–2019 and coverage in 2015 (the baseline year). Figure 4 present the results of correlation analysis between cumulative ODA per capita for water and sanitation in 2016–2019 and coverage in 2015 (the baseline year). Table 3 and Figure 4 show that overall, ODA per capita was not associated with baseline coverage (at least basic water and sanitation, log coefficient: 0.004, *p* = 0.17; safely managed water and sanitation, log coefficient: 0.005, *p* = 0.18).

## 4. Discussion

This study shows ODA has not been provided in accord with water and sanitation coverage, and that countries with the greatest need for water and sanitation were not prioritized for resource allocation during the 20 years from 2000 to 2019. Similarly, when the analysis was restricted to data for the SDGs period (2016–2019), aid was not disbursed in proportion to demand.

A supplementary but nevertheless meaningful finding of this study is that aid disbursements have fluctuated considerably; for example, although overall ODA for water and sanitation annual growth rate showed a slight increase from 2002, it remained constant or decreased from 2016.

We decided not to conduct a longitudinal analysis in this study since we found that there were no significant regression coefficients in most single-year analyses between the independent and dependent variables. We presented an analysis of results between the combined amount of ODA and the coverage of water and sanitation at baseline in 2015. Since there were no significant results in the regression analyses, we conducted a correlation analysis and found no correlation. We added the correlation analysis results to supplementary materials.

Our results are consistent with previous findings regarding the lack of a significant association between ODA per capita and water and sanitation coverage [24]. Our regression results show that donors did not allocate more ODA to recipient countries with lowest coverage during the 18-year period from 2002 to 2019, which indicates that ODA allocations for water and sanitation are moving in the wrong direction in terms of meeting SDGs (sustainable development goals).

Although the global health community has improved access to at least basic drinking water and sanitation in many countries, the overall increase in ODA masks inappropriate, and probably unfair, distributions of assistance. This weakness in aid allocation for water and sanitation impedes the achievement of increasing coverage and meeting SDGs [11,31].

According to the 2020 SDG report, the COVID-19 pandemic triggered a severe economic crisis, and this has markedly undermined progress toward meeting SDGs by placing 23 million more people below the poverty line [32,33]. During this crisis, we emphasize that access to safe water and sanitation must be secured.

We expected that ODA in water and sanitation would have increased after the outbreak of COVID-19 as the attention of the global community and many country leaders seems to have been drawn to the importance of water and sanitation to tackle the spread of infectious diseases, including COVID-19. However, we noticed that it substantially decreased in 2020 compared to 2019 based on the latest data from the OECD DAC CRS website. We infer that this change might have been due to the spike in development assistance for health related to infectious diseases in 2020. In particular, USD 13.7 billion was invested in tackling COVID-19 alone in 2020 [34]. Caution is needed when interpreting this finding, since we do not know whether this is just a short-term phenomenon, or whether this pattern would be observed in the long-term period. Further comparative research is warranted to explore recent trends in investments and ODA in water and sanitation during the COVID-19 pandemic.

Based on poor ODA targeting for water and sanitation revealed by this study, we appeal to the international community to pay more attention to the adequate allocation of ODA in proportion to demand. Given that improved access to water and sanitation and the spread of COVID-19 are inextricably linked, we believe it is appropriate to stress the importance of appropriate and fair aid allocation.

Priorities for aid allocation remain controversial. Some argue that ODA tends to be determined using criteria other than recipient country needs, such as geographic or political factors [35]. We established that ODA allocations for water and sanitation were not determined based on consideration of countries’ needs. The global community must determine the reasons for this discrepancy and means of addressing the situation. In addition, global efforts are required to assess how much investment is needed to achieve the goals of SDG6.

According to a recent report issued jointly by WHO and UNICEF, 3 out of every 10 people worldwide do not have access to personal washing facilities at home, which is essential for preventing the spread of COVID-19. Furthermore, it has been estimated that billions will lack access to safe water and sanitation at home in 2030 [11]. Therefore, to achieve the goal of providing safely managed water and sanitation for all, continued attention must be paid to securing more funding from the ODA and developing domestic financing mechanisms.

The present study has several limitations that warrant consideration. First, we only used CRS-reported data, which did not include data from global health initiatives, non-DAC bilateral donors, NGOs, or foundations. Second, we did not address the effects of variables such as governance, transparency, or the existence of effective government systems, which may have confounded results. Ensuring access to basic drinking water and sanitation is a great responsibility because access to basic water and sanitation is fundamental in terms of preventing global health crises, as has been adequately demonstrated by the COVID-19 pandemic [36]. Continuous monitoring of compliance with ODA targets would better enable the global community to make informed decisions regarding investment and policy development targeting poverty reduction. The variable “water coverage plus sanitation coverage” was included in this study primarily because of the limited categories of the OECD DAC CRS database. Another reason is that many donors are investing funds into integrated water and sanitation interventions. Similarly, many projects or programs are being undertaken under the umbrella of combined WASH interventions. We are still considering the possibility of collecting the raw data from each donor, which will be done in the follow-up study. We believe that this study will trigger the WASH community to conduct follow-up studies based on a more accurate and precise categorization of ODA investments. Similarly, if we had a raw dataset indicating accessibility of water or sanitation at the individual level, we would be able to estimate the combined coverage of water and sanitation. We could not obtain a raw dataset indicating accessibility of water and sanitation at the individual level for all the target countries included in this study. Nonetheless, we do acknowledge that simply adding water coverage to sanitation coverage has flaws, though we believe that a low value of this “water coverage plus sanitation coverage” variable might indicate more needs than a high value.

## 5. Conclusions

The present study shows the global community has not adequately allocated resources for water and sanitation. Concerted efforts are required to enable informed decisions regarding aid allocations that reflect the needs of recipient countries and accelerate progress toward SDG6. Furthermore, in countries where COVID-19 is rampant, those with the lowest water and sanitation coverages should receive the greatest consideration from the global community. Realignment of investments addressing these inequalities in ODA allocation for water and sanitation would also help avoid the global spread of infectious diseases such as COVID-19.

## Figures and Tables

**Table 1:** Official development assistance for total water and sanitation (2002–2019) and official development assistance for basic water and sanitation (2010–2019).

	Year	2002	2003	2004	2005	2006	2007	2008	2009	-	-
OECD CRS Code ^a^											
ODA ^b^ for water supply and sanitation, total (CRS code:140-I.4) ^c^		1359	1695	2006	3362	3144	2900	3610	3677		
Annual growth rate (%)			24.7	18.3	67.6	−6.5	−7.7	24.5	1.9		
ODA per capita for water supply and sanitation, total ^d^		0.216	0.263	0.309	0.517	0.475	0.416	0.455	0.507		
Annual growth rate (%)			21.6	17.6	67.2	−8.1	−12.3	9.2	11.4		
	Year	2010	2011	2012	2013	2014	2015	2016	2017	2018	2019
OECD CRS code											
ODA for water supply and sanitation, total (CRS code:140-I.4)		3990	4030	3790	3836	4125	4317	4336	4470	4349	3984
Annual growth rate (%)		8.5	1.0	−6.0	1.2	7.5	4.7	0.4	3.1	−2.7	−8.4
ODA per capita for water supply and sanitation, total		0.539	0.538	0.496	0.490	0.529	0.527	0.539	0.550	0.520	0.473
Annual growth rate (%)		6.3	−0.2	−7.7	−1.1	7.8	−0.2	2.2	2.0	−5.4	−9.0
ODA for basic drinking water supply (CRS code:14031)		860	833	838	944	882	956	943	858	829	812
Annual growth rate (%)			−3.1	0.6	12.6	−6.6	8.3	−1.3	−8.9	−3.3	−2.1
ODA per capita for basic drinking water supply		0.177	0.165	0.162	0.183	0.142	0.153	0.147	0.127	0.099	0.104
Annual growth rate (%)			−6.6	−2.0	12.6	−21.9	7.5	−3.8	−13.8	−21.7	4.9
ODA for basic sanitation (CRS code:14032)		740	811	777	888	851	848	816	797	815	722
Annual growth rate (%)			9.6	−4.2	14.3	−4.2	−0.2	−3.8	−2.2	2.1	−11.3
ODA per capita for basic sanitation		0.148	0.157	0.143	0.166	0.133	0.122	0.118	0.112	0.091	0.087
Annual growth rate (%)			5.8	−8.6	15.8	−19.6	−8.2	−3.4	−5.3	−18.5	−4.5

^a^ The Organisation for Economic Co-operation and Development (OECD) Creditor Reporting System (CRS) database does not contain full data before 2010. There are very restricted data before 2010, ^b^ ODA = official development assistance, ^c^ Annual trends ODA financing for water and sanitation coverage are in millions of 2019 USD, ^d^ ODA per capita is in 2019 USD.

**Table 2:** Associations between ODA and water and sanitation coverage in 2002, 2010, and 2019.

Regression Model					
Dependent	Independent	Coefficient	95% CI	*p*	R^2^
ln **(ODA per capita)**	**Water and sanitation coverage**				
water and sanitation, 2002	At least basic, 2002	0.003	0.001, 0.006	0.04	0.041
water and sanitation, 2010	At least basic, 2010	0.002	−0.001, 0.005	0.12	0.028
water and sanitation, 2019	At least basic, 2019	0.001	−0.002, 0.005	0.48	0.025
water and sanitation, 2002	Safely managed, 2002	0.002	−0.003, 0.006	0.52	0.012
water and sanitation, 2010	Safely managed, 2010	0.005	0.001, 0.009	0.03	0.063
water and sanitation, 2019	Safely managed, 2019	0.002	−0.003, 0.007	0.54	0.085
ln **(ODA per capita, basic)**	**Water coverage**				
basic water, 2010	At least basic, 2010	−0.001	−0.016, 0.013	0.87	0.001
basic water, 2019	At least basic, 2019	−0.002	−0.025, 0.019	0.81	0.059
basic water, 2010	Safely managed, 2010	−0.001	−0.018, 0.017	0.96	0.001
basic water, 2019	Safely managed, 2019	0.006	−0.012, 0.024	0.53	0.052
ln **(ODA per capita, basic)**	**Sanitation coverage**				
basic sanitation, 2010	At least basic, 2010	−0.005	−0.015, 0.003	0.24	0.016
basic sanitation, 2019	At least basic, 2019	−0.002	−0.016, 0.011	0.68	0.041
basic sanitation, 2010	Safely managed, 2010	0.002	−0.019, 0.024	0.79	0.002
basic sanitation, 2019	Safely managed, 2019	−0.011	−0.031, 0.008	0.28	0.061
Covariate: GNI per capita; ODA = official development assistance					

**Table 3:** Association between cumulated ODA per capita for water and sanitation in 2016–2019 and coverage in 2015.

ODA Targeting, MDGs to SDGs					
Dependent	Independent	Coefficient	95% CI	P	R^2^
ln (ODA per capita for water and sanitation), 2016–2019	At least basic water and sanitation coverage, GNI per capita, 2015	0.004	−0.001, 0.011	0.16	0.016
ln (ODA per capita for water and sanitation), 2016–2019	Safely managed water and sanitation coverage, GNI per capita, 2015	0.005	−0.002, 0.012	0.18	0.025
ln (ODA per capita for basic water), 2016–2019	At least basic water coverage, GNI per capita, 2015	−0.008	−0.034, 0.017	0.53	0.053
ln (ODA per capita for basic water), 2016–2019	Safely managed water coverage, GNI per capita, 2015	0.001	−0.024, 0.026	0.93	0.018
ln (ODA per capita for basic sanitation), 2016–2019	At least basic sanitation coverage, GNI per capita, 2015	−0.008	−0.025, 0.007	0.27	0.046
ln (ODA per capita for basic sanitation), 2016–2019	Safely managed sanitation coverage, GNI per capita, 2015	−0.018	−0.043, 0.007	0.16	0.055

MDGs = millennium development goal; SDGs = sustainable development goal; ODA = official development assistance.

## Data Availability

Not applicable.

## Supplementary Materials

The following supporting information can be downloaded at: https://www.mdpi.com/article/10.3390/ijerph191711134/s1, Table S1: Country code; Table S2: Associations between ODA per capita and coverage of water and sanitation.
