# Supplementary material for: Association between Official Development Assistance for Water and Sanitation and Countries’ Needs from 2002 to 2019
Source: Int J Environ Res Public Health. 2022 Sep 5;19(17):11134. doi: 10.3390/ijerph191711134 (PMC9518171; doi:10.3390/ijerph191711134)
Supplement: Supplementary file 1 [file ijerph-19-11134-s001.zip › ijerph-1812532-supplementary.pdf]

Table S1. Country code

Country (country code)		
Afghanistan(AF)	Democratic Republic of the Congo(CD)	
Albania(AL)	Djibouti(DJ)	Liberia(LR)
Algeria(DZ)	Dominica(DM)	Libya(LY)
Angola(AO)	Dominican Republic(DO)	Madagascar(MG)
	Ecuador(EC)	Malawi(MW)
Argentina(AR)	Egypt(EG)	Malaysia(MY)
Armenia(AM)	El Salvador(SV)	Maldives(MV)
Azerbaijan(AZ)	Equatorial Guinea(GQ)	Mali(ML)
Bangladesh(BD)	Eritrea(ER)	Marshall Islands(MH)
	Eswatini(SZ)	Mauritania(MR)
Belarus(BY)	Ethiopia(ET)	Mauritius(MU)
Belize(BZ)	Fiji(FJ)	Mexico(MX)
Benin(BJ)	Gabon(GA)	Micronesia(FM)
Bhutan(BT)	Gambia(GM)	Moldova(MD)
Bolivia(BO)	Georgia(GE)	Mongolia(MN)
Bosnia and Herzegovina(BA)	Ghana(GH)	Montenegro(ME)
Botswana(BW)	Grenada(GD)	Morocco(MA)
Brazil(BR)	Guatemala(GT)	Mozambique(MZ)
Burkina Faso(BF)	Guinea(GN)	Myanmar(MM)
Burundi(BI)	Guinea-Bissau(GW)	Namibia(NA)
Cabo Verde(CV)	Guyana(GY)	
Cambodia(KH)	Haiti(HT)	Nepal(NP)
Cameroon(CM)	Honduras(HN)	Nicaragua(NI)
Central African Republic(CF)	India(IN)	Niger(NE)
Chad(TD)	Indonesia(ID)	Nigeria(NG)
	Iran(IR)	North Macedonia(MK)
China(CN)	Iraq(IQ)	
Colombia(CO)	Jamaica(JM)	Pakistan(PK)
Comoros(KM)	Jordan(JO)	
Congo(CG)	Kazakhstan(KG)	Panama(PA)
Costa Rica(CR)	Kenya(KE)	Papua New Guinea(PG)
Côte d'Ivoire(CI)	Kiribati(KI)	Paraguay(PY)
	Kyrgyzstan(KG)	Peru(PE)
Cuba(CU)	Lao People's Democratic Republic(LA)	Philippines(PH)
Democratic People's Republic of Korea(KP)	Lebanon(LB)	Rwanda(RW)
	Lesotho(LS)	Samoa(WS)

		Sao Tome and Principe(ST)
Senegal(SN)		
Serbia(RS)		
Sierra Leone(SL)		
Solomon Islands(SB)		
Somalia(SO)		
South Africa(ZA)		
South Sudan(SS)		
Sri Lanka(LK)		
Sudan(SD)		
Suriname(SR)		
Syrian Arab Republic(SY)		
Tajikistan(TJ)		
Tanzania(TZ)		
Thailand(TH)		
Timor-Leste(TL)		
Togo(TG)		
Tonga(TO)		
Tunisia(TN)		
Turkey(TR)		
Turkmenistan(TM)		
Tuvalu(TV)		
Uganda(UG)		
Ukraine(UA)		
Uzbekistan(UZ)		
Vanuatu(VU)		
Venezuela(VE)		
Viet Nam(VN)		
West Bank and Gaza Strip(PS)		
Yemen(YE)		
Zambia(ZM)		
Zimbabwe(ZW)		

Table S2. Associations between ODA per capita and coverage of water and sanitation

**Correlation**			
**Dependent**	**Independent**	***r***	***p***
ln **(ODA per capita, basic)**	**Water coverage**		
basic water, 2010	At least basic, 2010	-0.016	0.87
basic water, 2011	At least basic, 2011	-0.039	0.70
basic water, 2012	At least basic, 2012	-0.020	0.84
basic water, 2013	At least basic, 2013	0.008	0.93
basic water, 2014	At least basic, 2014	-0.022	0.82
basic water, 2015	At least basic, 2015	-0.044	0.66
basic water, 2016	At least basic, 2016	-0.104	0.30
basic water, 2017	At least basic, 2017	-0.089	0.39
basic water, 2018	At least basic, 2018	-0.153	0.14
basic water, 2019	At least basic, 2019	-0.182	0.08
basic water, 2010	Safely managed, 2010	-0.024	0.86
basic water, 2011	Safely managed, 2011	-0.101	0.48
basic water, 2012	Safely managed, 2012	-0.011	0.93
basic water, 2013	Safely managed, 2013	0.011	0.94
basic water, 2014	Safely managed, 2014	-0.013	0.93
basic water, 2015	Safely managed, 2015	-0.018	0.89
basic water, 2016	Safely managed, 2016	-0.041	0.76
basic water, 2017	Safely managed, 2017	-0.028	0.84
basic water, 2018	Safely managed, 2018	-0.011	0.94
basic water, 2019	Safely managed, 2019	-0.068	0.62
ln **(ODA per capita, basic)**	**Sanitation coverage**		
basic sanitation, 2010	At least basic, 2010	-0.127	0.22
basic sanitation, 2011	At least basic, 2011	-0.146	0.16
basic sanitation, 2012	At least basic, 2012	-0.062	0.56
basic sanitation, 2013	At least basic, 2013	-0.068	0.49
basic sanitation, 2014	At least basic, 2014	-0.107	0.29
basic sanitation, 2015	At least basic, 2015	-0.081	0.44
basic sanitation, 2016	At least basic, 2016	-0.132	0.20
basic sanitation, 2017	At least basic, 2017	-0.153	0.14
basic sanitation, 2018	At least basic, 2018	-0.175	0.09
basic sanitation, 2019	At least basic, 2019	-0.174	0.09
basic sanitation, 2010	Safely managed, 2010	0.038	0.79
basic sanitation, 2011	Safely managed, 2011	-0.132	0.35

basic sanitation, 2012	Safely managed, 2012	-0.135	0.36
basic sanitation, 2013	Safely managed, 2013	-0.184	0.18
basic sanitation, 2014	Safely managed, 2014	-0.191	0.17
basic sanitation, 2015	Safely managed, 2015	-0.183	0.18
basic sanitation, 2016	Safely managed, 2016	-0.206	0.13
basic sanitation, 2017	Safely managed, 2017	-0.224	0.11
basic sanitation, 2018	Safely managed, 2018	-0.207	0.14
basic sanitation, 2019	Safely managed, 2019	-0.199	0.16

Covariate: GNI per capita; ODA=official development assistance
